# Derivation, validation, and comparison of a new prognostic scoring system for acute lower gastrointestinal bleeding

**DOI:** 10.1002/deo2.323

**Published:** 2023-12-11

**Authors:** Christopher Smith, Gillian Leggett, Anthoor Jayaprakash, Mohammed Khan, John M. Thomson, Balasubramaniam Vijayan, Andrew Fraser, John S. Leeds

**Affiliations:** ^1^ Department of Gastroenterology Aberdeen Royal Infirmary Aberdeen UK; ^2^ Department of Gastroenterology Northumbria Healthcare Cramlington UK; ^3^ Department of Gastroenterology Queen Elizabeth University Hospital Glasgow UK; ^4^ Population Health Sciences Institute Newcastle University Newcastle Upon Tyne UK

**Keywords:** lower intestinal bleeding, morbidity, mortality, prognosis, rebleeding

## Abstract

**Objectives:**

Lower gastrointestinal bleeding is a common presentation with little data concerning risk factors for adverse outcomes. The aim was to derive and validate a scoring system to stratify risk in lower gastrointestinal bleeding and compare it to the Oakland score.

**Methods:**

A total of 2385 consecutive patients (mean age 65 years, 1140 males) were used to derive the score using multivariate logistic regression modeling then internally and externally validated. The Oakland score was applied and area under receiver operating characteristic (AUROC) curves were calculated and compared. A score of <1 was compared with an Oakland score of <9 to assess 30‐day rebleeding and mortality rates.

**Results:**

Rebleeding was associated with age, inpatient bleeding, syncope, malignancy, tachycardia, hypotension, lower hemoglobin and mortality with age, inpatient bleeding, liver/gastrointestinal disease, tachycardia, and hypotension. The area under the receiver operating characteristic curves was 0.742 for rebleeding and 0.802 for mortality. A score <1 was associated with rebleeding (0.0%–2.2%) and mortality (0%). The Oakland score had a significantly lower area under the receiver operating characteristic curve for rebleeding of 0.687 but not for mortality; 0.757. A score <1 was associated with a lower 30‐day rebleeding risk compared to an Oakland score <9 (4/379 vs. 15/355, *p* = 0.009) but not mortality (0/365 vs. 1/355, *p* = 0.493).

**Conclusions:**

Our score predicts 30‐day rebleeding and mortality rate with low scores associated with very low risk. The Aberdeen score is superior to the Oakland score for predicting rebleeding. Prospective evaluation of both scores is required.

## INTRODUCTION

Lower gastrointestinal bleeding (LGIB) has an incidence between 33–412/100,000 population representing a significant healthcare burden.^1^, ^2^ The UK audit showed the majority of patients required no intervention to identify and treat the bleeding source indicating outpatient management may be appropriate.^3^ LGIB is generally less severe than upper gastrointestinal bleeding with most cases resolving spontaneously.^4^ Patients with LGIB are often older, comorbid, and on anticoagulants.^3^ Risk stratification scores are utilized to aid decision making^5^ and several are available for upper gastrointestinal bleeding.^6^, ^7^ The ‘holy grail’ would be determining the need for intervention or who could safely managed as an outpatient optimising resources.^3^ Previous studies identified risk factors for adverse outcomes in LGIB or used upper intestinal scoring systems but failed to provide a workable score.^8^, ^9^, ^10^ A new score was proposed to predict safe discharge in patients with LGIB using readily available variables with a score <9 associated with a 95% chance of safe discharge.^11^ This was only if the patient had no other reasons for admission. The Oakland score has been incorporated into national guidelines^12^ recommending patients with a score >8 undergo urgent inpatient colonoscopy which lacks supporting evidence.^13^, ^14^, ^15^, ^16^

We analyzed our bleeding unit database to derive and validate a risk prediction model for patients admitted with acute LGIB and compare it with the Oakland score.

## PATIENTS AND METHODS

The Aberdeen Royal Infirmary Gastrointestinal Bleeding Unit opened in October 1991 for patients presenting with suspected acute intestinal bleeding and serves a population of approximately 650,000. Patients were admitted directly to the unit and managed with standardized patient assessment, focusing on resuscitation and timely endoscopy. Admissions were prospectively entered into the bleeding unit database. Admission variables included demographics, admission source, bleeding symptoms, medications including anticoagulants, smoking history, alcohol history, and past medical and surgical history. Information concerning comorbidities was collected and graded from 0–3 depending on clinical status (Document SA). Admission supine systolic blood pressure (mmHg), pulse rate (beats/minute), hemoglobin (Hb, g/dL), and urea were measured and recorded. Data was recorded on blood transfusion, endoscopic findings, surgical intervention, occurrence of rebleeding, and death within 30 days. Rebleeding was defined as either the commencement of new bleeding following initial hemostasis, a fall in Hb >2g/dL following initial hemostasis, new hemodynamic instability, or a combination of these factors.^8^, ^11^

### Inclusion and exclusion criteria

Inclusion criteria were presentation with hematochezia or melena in the presence of hematochezia. Patients with fresh/altered hematemesis were excluded. Patients that had been discharged and readmitted with rebleeding within 30 days were classified as “rebleed”. Those readmitted after more than 30 days were classified as new cases.

### Study cohorts

The derivation cohort comprised individuals admitted from 1991 to 2006. Endoscopic intervention is rare in LGIB and has not changed in the UK over this time.^3^ The internal validation cohort comprised unselected admissions for the period 2015–2016. External validation was conducted using a cohort of unselected patients admitted to the Northumbria Specialist Emergency Care Hospital, Cramlington, UK from 2015 to 2018.

### Outcome measures

30‐day outcome measures were rebleeding and death. Factors associated with blood transfusion and surgical intervention were analyzed as secondary outcome measures as both require additional, clinical decision‐making and are not hard endpoints.

### Statistical analysis and score modeling

Data analysis was undertaken using Statistical Package for the Social Sciences version 19.0. Univariate analysis for 30‐day rates of blood transfusion, rebleeding, surgical intervention, and death and expressed as odds ratios with 95% confidence intervals. Patients with missing variables were excluded. Variables with a *p*‐value <0.1 were entered into a multiple logistic regression analysis to identify independent risk factors for each outcome by using a forward stepwise selection procedure. Final variables were analyzed by binary logistic regression to generate logistic regression coefficients, *p*‐values, adjusted odds ratios, and 95% confidence intervals.

Prognostic scoring systems (the Aberdeen score) were derived using relative weightings based on the integers obtained from multivariable analysis. Relative weightings were assigned to clinically meaningful sub‐categorizations; with reference parameters assigned the lowest weighting, and the highest risk categories assigned the highest weighting.^17^

Initial validation was performed using risk stratification, discriminative capacity, and calibration analysis. Internal and external validation was then performed.

Threshold analysis was performed to determine whether the scoring systems were able to determine which patients were at the lowest risk for adverse outcomes.^6^

Finally, the Oakland score^11^ was calculated for the derivation cohort and compared with the Aberdeen score. This score has a predefined cut‐off of <9 as being suitable for outpatient management and receiver operating characteristic (ROC) curves were used to compare 30‐day rebleeding and mortality rates. A comparison of the different ROC curves was performed using the Delong test.

### Ethical considerations

This study was approved by the North of Scotland Research Ethics Service (reference number: 11/NS/0043, 2011). As this was a retrospective analysis of an institutional database there was no clinical trial registry or retrospective consent.

## RESULTS

Note that, 2385 consecutive patients were admitted to the Aberdeen bleeding unit with acute LGIB (mean age 65 years, 1140 males). Table 1 shows the admission characteristics of the derivation cohort. All patients presented with fresh rectal bleeding without hematemesis or coffee‐ground vomiting. Melena was reported in 592/2385 (24.8%) prior to admission while 740/2385 (31%) presented with concomitant diarrhea. 751/2385 (31.5%) reported ≥1 previous episode of gastrointestinal bleeding. Cardiovascular disease was the most prevalent comorbidity reported in 847 (35.5%) followed by arthritis (22.0%) and stroke/neurological conditions (12.3%). 443/2385 (18.6%) were tachycardic, 173/2385 (7.3%) were hypotensive and 72/2385 (3.0%) were both tachycardic and hypotensive on admission. 143/2385 (6.0%) described syncope during assessment. 228/2385 (9.6%) were taking anticoagulants and 684/2385 (28.7%) were taking aspirin.

**TABLE 1:** Derivation cohort patient characteristics.

Variable	Categorization	Frequency	Percentage (%)
Sex	Male	1140	47.8
	Female	1245	52.2
Age group	<60	758	31.8
	60–79	1013	42.5
	≥80	614	25.7
Admission source	General practice	1716	71.9
	Accident and emergency	195	8.2
	Current inpatient in the same hospital	287	12
	Other hospital	180	7.5
	*Missing*	7	0.3
Admission source	Primary care	1911	80.1
	Secondary care	467	19.6
	*Missing*	7	0.3
Fresh PR bleeding	No	0	0
	Yes	2385	100
Melena	No	1793	75.2
	Yes	592	24.8
Syncope	No	1722	72.2
	Yes	142	6.0
	*Missing*	521	21.8
Diarrhea	No	1613	67.6
	Yes	740	31.0
	*Missing*	32	1.3
Anti‐ulcer therapy	No	1754	73.5
	Yes	628	26.3
	*Missing*	3	0.1
Anti‐coagulation therapy	No	2145	90.3
	Yes	228	9.6
	*Missing*	3	0.1
Aspirin use	No	1699	71.2
	Yes	684	28.7
	*Missing*	2	0.1
Other nsaid use	No	1957	82.1
	Yes	424	17.8
	*Missing*	4	0.2
Total no. of regular medications	0	285	11.9
	<5	837	35.1
	≥5	726	30.4
	*Missing*	537	22.5
Tobacco use	No	1793	75.2
	Yes	584	24.5
	*Missing*	8	0.3
Alcohol use	No	1000	41.9
	Yes	864	36.2
	*Missing*	521	21.8
Past gi bleed	No	1629	68.3
	Yes	751	31.5
	*Missing*	5	0.2
Past gastric ulcer	No	2284	95.8
	Yes	95	4
	*Missing*	6	0.3
Past duodenal ulcer	No	2154	90.3
	Yes	224	9.4
	*Missing*	7	0.3
Past esophageal varices	No	1831	76.8
	Yes	32	1.3
	*Missing*	522	21.9
Liver/GI	0	1526	64
	1	176	7.4
	2	104	4.4
	3	41	1.7
	*Missing*	538	22.6
Cardiovascular	0	1000	41.9
	1	559	23.4
	2	221	9.3
	3	67	2.8
	*Missing*	538	22.6
Respiratory	0	1452	60.9
	1	216	9.1
	2	109	4.6
	3	26	1.1
	*Missing*	582	24.4
Arthritis	0	1276	53.5
	1	415	17.4
	2	72	3
	3	39	1.6
	*Missing*	583	24.4
Stroke/neurology	0	1507	63.2
	1	211	8.8
	2	60	2.5
	3	25	1
	*Missing*	582	24.4
Renal	0	1695	71.1
	1	59	2.5
	2	34	1.4
	3	14	0.6
	*Missing*	583	24.4
Malignancy	0	1583	66.4
	1	34	1.4
	2	142	6
	3	43	1.8
	*Missing*	583	24.4
Heart rate	<100	1911	80.1
	≥100	443	18.6
	*Missing*	31	1.3
Supine systolic blood pressure	≥100	2183	91.5
	<100	173	7.3
	*Missing*	29	1.2
Hemoglobin (g/dL)	≥14	706	29.6
	10–<14	1204	50.5
	<10	472	19.8
	*Missing*	3	0.1
Urea (g/dL)	<10	1758	73.7
	≥10	607	25.5
	*Missing*	20	0.8
Received blood transfusion	0	1514	63.5
	<5 units	666	27.9
	≥5 units	202	8.5
	*Missing*	3	0.1
Rebleeding	No	2059	86.3
	Yes	322	13.5
	*Missing*	4	0.2
Surgical intervention	No	2250	93.5
	Yes	135	5.7
Mortality at 30 Days	No	2256	94.4
	Yes	129	5.6

868 (36.4%) patients required blood transfusion, 202 (8.5%) of whom required ≥5 units. Three hundred twenty‐two (13.5%) experienced rebleeding, 135 (5.7%) required surgical intervention, and 129 (5.6%) died within 30 days of admission.

### Univariate and multivariable analyses

Table 2 shows univariate and multivariable analyses for 30‐day rebleeding and mortality. On univariate analysis rebleeding was associated with increasing age, inpatient bleeding episodes (secondary care), melena, syncope, aspirin use, increasing number of medications, previous gastrointestinal bleeding, previous stroke/neurological disease, underlying malignancy, tachycardia, hypotension, lower Hb, and raised urea level. Presenting with diarrhea, smoking, and absence of respiratory disease were all protective against rebleeding. Multivariable analysis showed rebleeding was independently associated with increasing age (odds ratio [OR] 1.54 for 60–79 years and 2.07 for ≥80 years), inpatient bleeding episode (OR 2.66), syncope (OR 3.26), underlying malignancy (grade 2 OR 2.10 and grade 3 OR 4.54), tachycardia (OR 1.7), hypotension (OR 3.47) and decreasing Hb (10–13.9 OR 2.53 and <10 OR 4.99).

**TABLE 2:** Univariate and multivariate analyses in the derivation cohort.

	Rebleeding		Mortality	
Variable	Unadjusted OR (95% CI)	Adjusted OR (95% CI)	Unadjusted OR (95% CI)	Adjusted OR (95% CI)
**Sex**				
Male	Reference		Reference	
Female	0.81 (0.64–1.02) *p* = 0.700		0.87 (0.61–1.24) *p* = 0.432	
**Age group**				
<60	Reference	Reference	Reference	Reference
60–79	1.96 (1.43–2.69) *p* < 0.001	1.54 (1.00–2.35) *p* = 0.048	3.17 (1.79–5.63) *p* < 0.001	3.28 (1.51–7.13) *p* = 0.003
≥80	2.71 (1.95–3.78) *p* < 0.001	2.07 (1.33–3.22) *p* = 0.001	4.68 (2.61–8.39) *p* < 0.001	6.01 (2.63–13.73) *p* < 0.001
**Admission source**				
Primary care	Reference	Reference	Reference	Reference
Secondary care	2.66 (2.06–3.44) *p* < 0.001	1.83 (1.32–2.54) *p* < 0.001	4.83 (3.36–6.93) *p* < 0.001	3.29 (2.01–5.40) *p* < 0.001
**Melena**				
No	Reference		Reference	
Yes	1.35 (1.04–1.75) *p* = 0.024		1.56 (1.07–2.27) *p* = 0.022	
**Syncope**				
No	Reference	Reference	Reference	
Yes	3.26 (2.19–4.86) *p* < 0.001	2.31 (1.49–3.59) *p* < 0.001	2.49 (1.34–4.63) *p* = 0.004	
**Diarrhea**				
No	Reference		Reference	
Yes	0.65 (0.50–0.86) *p* = 0.002		0.56 (0.36–0.87) *p* = 0.009	
**Anti‐ulcer therapy**				
No	Reference		Reference	
Yes	1.18 (0.91–1.52) *p* = 0.221		1.37 (0.94–2.01) *p* = 0.102	
**Anti‐coagulation therapy**				
No	Reference		Reference	
Yes	1.37 (0.95–1.98) *p* = 0.093		2.56 (1.63–4.04) *p* < 0.001	
**Aspirin use**				
No	Reference		Reference	
Yes	1.33 (1.03–1.70) *p* = 0.027		0.85 (0.56–1.27) *p* = 0.421	
**Other NSAID use**				
No	Reference		Reference	
Yes	1.17 (0.87–1.58) *p* = 0.289		1.06 (0.67–1.67) *p* = 0.808	
**No. of medications**				
0	Reference		Reference	
1–4	1.26 (0.77–2.07) *p* = 0.352		0.60 (0.26–1.37) *p* = 0.223	
≥5	2.28 (1.41–3.68) *p* = 0.001		2.42 (1.18–4.69) *p* = 0.016	
**Smoking**				
No	Reference		Reference	
Yes	0.67 (0.50–0.90) *p* = 0.009		0.85 (0.56–1.31) *p* = 0.467	
**Alcohol use**				
No	Reference		Reference	
Yes	1.06 (0.80–1.41) *p* = 0.669		0.68 (0.43–1.09) *p* = 0.106	
**Past GI bleed**				
No	Reference		Reference	
Yes	1.52 (1.19–1.93) *p* = 0.001		0.80 (0.54–1.19) *p* = 0.267	
**Past GU**				
No	Reference		Reference	
Yes	0.74 (0.38–1.45) *p* = 0.381		0.97 (0.39–2.43) *p* = 0.944	
**Past DU**				
No	Reference		Reference	
Yes	0.90 (0.60–1.37) *p* = 0.627		0.72 (0.36–1.43) *p* = 0.344	
**Past varices**				
No	Reference		Reference	
Yes	0.78 (0.24–2.57) *p* = 0.679		2.36 (0.70–7.91) *p* = 0.165	
**Liver/GI grade**				
0	Reference		Reference	Reference
1	1.15 (0.72–1.83) *p* = 0.553		0.45 (0.14–1.44) *p* = 0.178	0.45 (0.13–1.53) *p* = 0.202
2	1.00 (0.54–1.86) *p* = 0.998		2.74 (1.36–5.54) *p* = 0.005	3.58 (1.65–7.75) *p* = 0.001
3	1.58 (0.69–3.61) *p* = 0.281		8.31 (3.89–17.78) *p* < 0.001	7.18 (2.91–17.73) *p* < 0.001
**Cardiovascular grade**				
0	Reference		Reference	
1	1.34 (0.98–1.84) *p* = 0.070		1.08 (0.62–1.87) *p* = 0.794	
2	1.25 (0.80–1.95) *p* = 0.326		2.01 (1.08–3.74) *p* = 0.028	
3	1.69 (0.86–3.33) *p* = 0.128		4.28 (1.96–9.32) *p* < 0.001	
**Respiratory grade**				
0	Reference		Reference	
1	0.51 (0.29–0.88) *p* = 0.015		7.91 (0.36–1.75) *p* = 0.564	
2	0.85 (0.46–1.58) *p* = 0.608		2.13 (1.02–4.41) *p* = 0.043	
3	0.89 (0.26–2.98) *p* = 0.847		1.97 (0.45–8.52) *p* = 0.366	
**Arthritis grade**				
0	Reference		Reference	
1	1.20 (0.86–1.67) *p* = 0.285		1.16 (0.67–2.02) *p* = 0.599	
2	1.42 (0.73–2.75) *p* = 0.306		1.11 (0.34–3.66) *p* = 0.861	
3	1.43 (0.59–3.46) *p* = 0.432		6.60 (2.88–15.13) *p* < 0.001	
**Stroke/neurology grade**				
0	Reference		Reference	
1	1.26 (0.83–1.92) *p* = 0.286		1.61 (0.87–2.99) *p* = 0.131	
2	1.57 (0.78–3.16) *p* = 0.204		1.29 (0.39–4.25) *p* = 0.673	
3	3.06 (1.26–7.42) *p* = 0.014		2.13 (0.49–9.27) *p* = 0.312	
**Renal grade**				
0	Reference		Reference	
1	1.78 (0.91–3.49) *p* = 0.094		1.28 (0.39–4.20) *p* = 0.682	
2	1.31 (0.50–3.42) *p* = 0.582		1.50 (0.35–6.37) *p* = 0.586	
3	2.07 (0.57–7.49) *p* = 0.266		9.57 (2.93–31.29) *p* < 0.001	
**Malignancy grade**				
0	Reference	Reference	Reference	
1	1.82 (0.74–4.45) *p* = 0.192	1.31 (0.50–3.42) *p* = 0.579	1.53 (0.36–6.54) *p* = 0.564	
2	2.10 (1.35–3.27) *p* = 0.001	1.72 (1.08–2.75) *p* = 0.024	1.08 (0.46–2.55) *p* = 0.856	
3	4.54 (2.38–8.67) *p* < 0.001	2.09 (1.02–4.28) *p* = 0.045	4.77 (2.04–11.14) *p* < 0.001	
**Heart rate**				
<100 beats per minute	Reference		Reference	Reference
≥100 beats per minute	1.70 (1.30–2.24) *p* < 0.001		2.49 (1.70–3.65) *p* < 0.001	2.10 (1.25–3.55) *p* = 0.005
**Supine systolic blood pressure**				
≥100 mmHg	Reference	Reference	Reference	Reference
<100 mmHg	3.47 (2.46–4.90) *p* < 0.001	2.26 (1.43–3.57) *p* < 0.001	4.70 (3.03–7.28) *p* < 0.001	2.86 (1.54–5.28) *p* = 0.001
**Hemoglobin (g/dL)**				
≥14	Reference	Reference	Reference	
10–13.9	2.53 (1.78–3.60) *p* < 0.001	3.11 (1.79–5.39) *p* < 0.001	2.54 (1.44–4.50) *p* = 0.001	
<10	5.08 (3.48–7.40) *p* < 0.001	4.99 (2.80–8.90) *p* < 0.001	5.58 (3.10–10.05) *p* < 0.001	
**Urea (g/dL)**				
<10	Reference		Reference	
≥10	2.40 (1.87–3.06) *p* < 0.001		5.22 (3.61–7.55) *p* < 0.001	

30‐day mortality was associated with increasing age, inpatient bleeding episodes, melena, syncope, anti‐coagulant use, increasing number of medications, underlying liver/gastrointestinal disease, underlying cardiovascular disease, underlying respiratory disease, advanced rheumatological disease, advanced renal disease, underlying malignancy, tachycardia, hypotension, lower Hb and raised urea levels. Presenting with diarrhea was protective against mortality at 30 days. Multivariable analysis showed mortality was independently associated with increasing age (OR 3.28 for 60–79 years and 6.01 for ≥80 years), inpatient bleeding episode (OR 3.29), underlying liver/gastrointestinal disease (grade 2 OR 3.58 and grade 3 OR 7.18), tachycardia (OR 2.1) and hypotension (OR 2.86).

### Surgery and blood transfusion

Surgery and blood transfusion produced fewer significant associations upon univariate analysis (Document SB). Blood transfusion was associated with increasing age, female sex, hypotension, and lower Hb. Being under 60 years of age and Hb >14 was protective against transfusion. Surgical intervention was associated with inpatient bleeding episodes, melena, syncope, NSAID use, hypotension, tachycardia, and lower Hb.

### Scoring system derivation

The regression coefficients used were from the multivariate analysis of the final re‐bleeding and mortality models. Table 3 shows both the rebleeding and mortality scores with the relative variable weightings. For rebleeding, only the lowest level of Hb (<10g/dL) contributed 2 points, while the remaining variables contributed either 0 or 1. The composite score ranged from 0 to 7.

**TABLE 3:** Rebleeding and mortality scores.

	**Rebleeding score**		
Variable	0	1	2
Age	<79	≥80	
Admission source	New admission	Inpatient	
Syncope	No	Yes	
Systolic blood pressure (mmHg)	≥100	<100	
Hemoglobin (g/dL)	≥14	<14–≥10	<10
Cancer comorbidity	None	Grade 2/3	
	**Mortality score**		
Variable	0	1	2
Age	<60	60–79	≥80
Admission source	New admission	Inpatient	
Heart rate (bpm)	<100	≥100	
Systolic blood pressure (mmHg)	≥100	<100	
Hepatic comorbidity	None	Grade 2	Grade 3

For mortality score, age and hepatic/GI co‐morbidity contribute 0–2 points, whereas admission source, heart rate, and blood pressure contribute a maximum of 1. The composite score ranged from 0 to 7.

### Initial score validation

**FIGURE 1:**
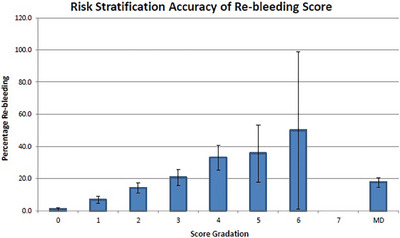
Risk stratification accuracy for the Rebleeding score in the derivation cohort.

**FIGURE 2:**
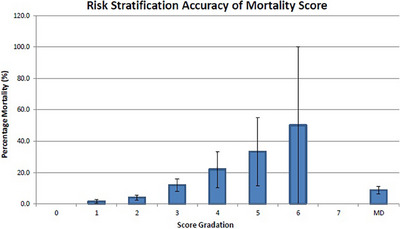
Risk stratification accuracy for the Mortality score in the derivation cohort.

Application of the scores to the derivation cohort showed a graded increase in the proportion of adverse events with increasing scores (Figures 1 and 2). Table 4 shows the full stratifications. For rebleeding. ROC analysis revealed an area under the curve (AUC) of 0.742 (0.709–0.774, *p* < 0.001). For mortality, ROC analysis revealed an AUC of 0.802 (0.755–0.848, *p* < 0.001). The Hosmer‐Lemeshow test for rebleeding and mortality showed good calibration for both rebleeding (*p* = 0.212) and mortality (*p* = 0.896; Documents SC and SD). A rebleeding score of 0 had a sensitivity of 98.8%, specificity of 15.8%, positive predictive value of 13.8%, and negative predictive value of 98.9%. A mortality score of 0 had a sensitivity of 100%, specificity of 15.3%, positive predictive value of 6.0%, and negative predictive value of 100%.

**TABLE 4:** Risk stratification of the rebleeding and mortality scores in all three cohorts.

	Rebleeding			Mortality		
Score	Total *N*	Number rebleeding	% Rebleeding (95% confidence interval)	Total *N*	Number died	% Died (95% confidence interval)
**Derivation cohort**						
0	379	4	1.1 (0.3–2.8)	365	0	0.0 (0.0–0.9)
1	509	35	6.9 (4.9–9.4)	592	10	1.7 (0.9–3.1)
2	474	67	14.1 (11.3–17.6)	532	21	3.9 (2.6–6.0)
3	241	50	20.7 (16.1–26.3)	243	29	11.9 (8.1–16.7)
4	139	46	33.1 (25.8–41.3)	50	11	22.0 (12.6–35.4)
5	28	10	35.7 (20.6–54.3)	18	6	33.3 (13.3–59.0)
6	4	2	50.0 (15.0–85.0)	2	1	50.0 (1.3–98.7)
7	0	0	0.0 (n/a)	0	0	0.0 (n/a)
Missing	611	110	18.0 (15.2–21.2)	583	51	8.7 (6.6–11.3)
**Internal validation cohort**						
0	32	0	0.0 (0.0–9.3)	35	0	0.0 (0.0–8.6)
1	55	3	5.5 (1.3–15.4)	54	3	5.5 (1.3–15.7)
2	18	2	11.1 (1.9–35.4)	28	1	3.6 (0.0–19.2)
3	12	2	16.7 (3.5–46.0)	3	0	0.0 (0.0–29.3)
4	3	0	0.0 (0.0–29.3)	1	1	100.0 (22.4–100.0)
5	1	0	0.0 (0.0–77.7)	0	n/a	‐
6	0	n/a	‐	0	n/a	‐
7	0	n/a	‐	0	n/a	‐
Missing	0	n/a	‐	0	n/a	‐
**External validation cohort**						
0	39	1	2.6 (0.0–14.4)	39	0	0.0 (0.0–7.8)
1	66	7	10.6 (4.4–20.6)	79	4	5.1 (1.6–12.7)
2	62	9	14.5 (7.6–25.6)	72	3	4.2 (0.9 ‐ 12.0)
3	30	3	10.0 (2.7–26.4)	22	6	27.3 (12.9–48.4)
4	14	2	14.3 (2.8–41.2)	1	0	0.0 (0.0–7.7)
5	1	0	0.0 (0.0–7.7)	0	n/a	‐
6	0	0	‐	0	0	‐
7	0	0	‐	0	0	‐
Missing	0	0	‐	0	0	‐

A rebleeding score of 0 was associated with a 1.1% (0.3–2.8) risk of rebleeding at 30 days. A mortality score of 0 was associated with a 0.0% (0.0–0.9) risk of death at 30 days.

### Internal validation

The internal validation cohort comprised 121 individuals (median age 68, 61 females) with 30‐day rebleeding and mortality rates of 7/121 (5.8%) and 5/121 (4.1%) respectively. Threshold analysis showed a score of 0 for rebleeding or for mortality was associated with a rebleeding and mortality rate of 0% at 30 days.

### External validation

The external validation cohort comprised 212 individuals (median age 72, 115 females) with 30‐day rebleeding and mortality rates of 22/212 (10.4%) and 13/212 (6.1%) respectively. Threshold analysis showed that a score of 0 for rebleeding was associated with a rebleeding rate of 2.6% at 30 days and a score of 0 for mortality was associated with a mortality rate of 0% at 30 days.

### Comparison with the Oakland score

**FIGURE 3:**
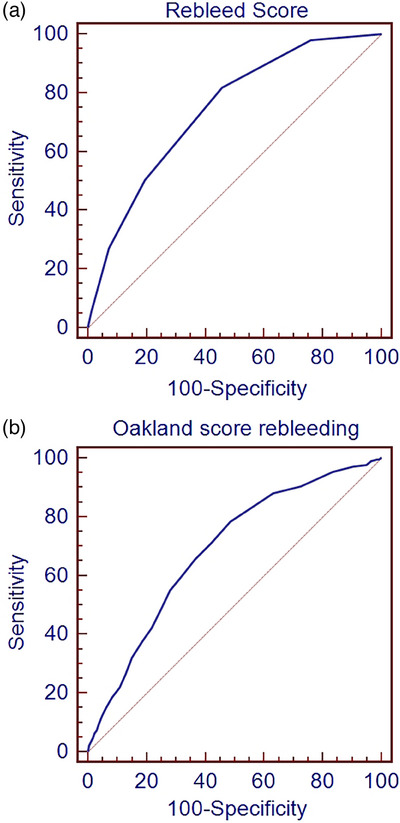
Receiver operating characteristic curves for the Aberdeen score (a) and the Oakland score (b) for rebleeding in the derivation cohort.

**FIGURE 4:**
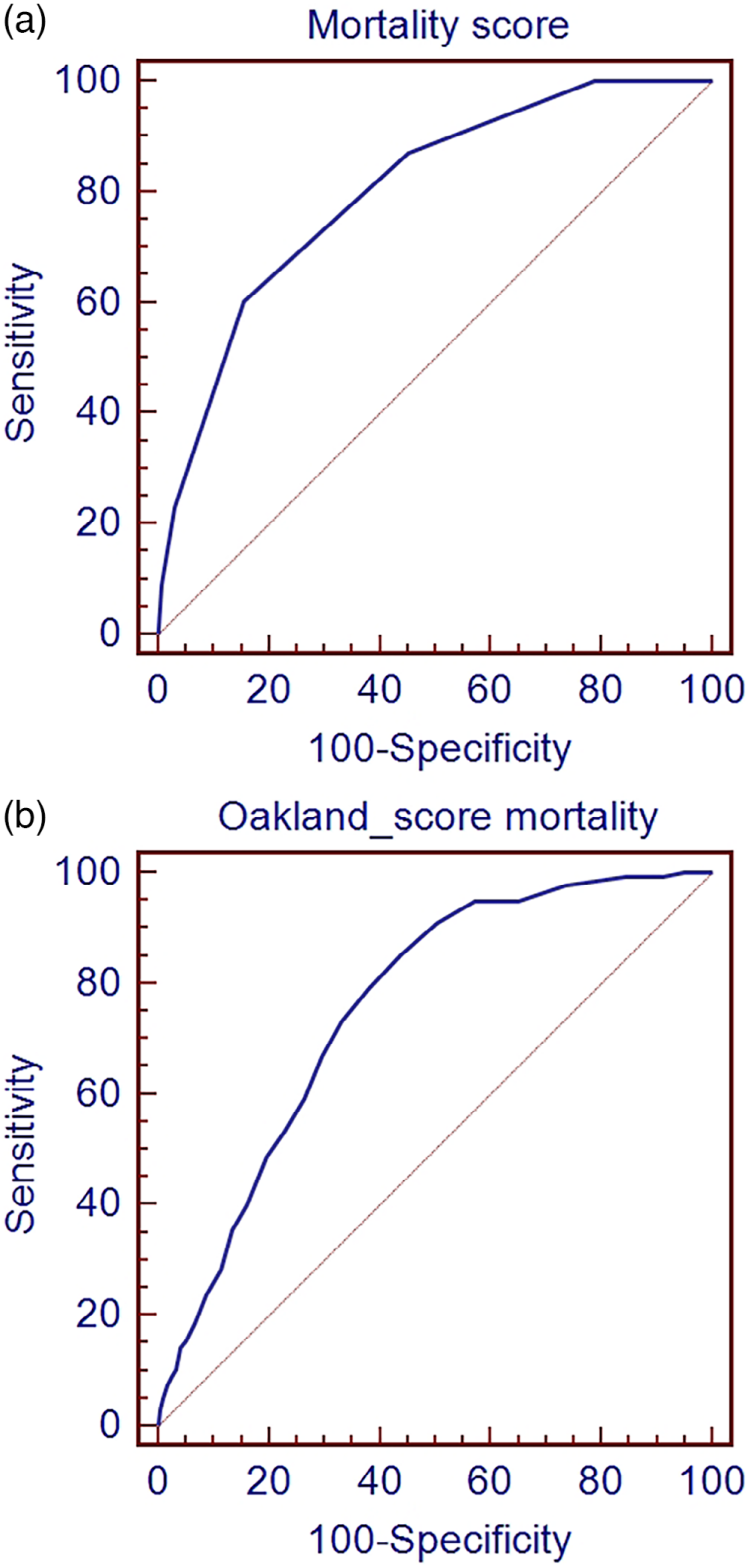
Receiver operating characteristic curves for the Aberdeen score (a) and the Oakland score (b) for mortality in the derivation cohort.

The Oakland score was calculated for the derivation cohort and ROC analysis was performed for rebleeding and mortality. The Oakland score had a significantly lower AUC than the rebleeding score (0.687 [0.668–0.705] vs. 0.742 [0.709–0.774] [Figure 3]). The Oakland score and the mortality score were not significantly different (0.757 [0.739–0.774] vs. 0.802 [0.755–0.848] [Figure 4]). Comparison of 30‐day rebleeding and mortality rates in patients with low scores (Aberdeen score <1 vs. Oakland score <9) showing rebleeding, 15/355 (4.2%) with an Oakland score <9 experienced rebleeding at 30 days compared to 4/379 (1.1%) with an Aberdeen score <1 (*p* = 0.009). For mortality, 1/355 (0.3%) with an Oakland score <9 had died at 30 days compared to 0/365 (0.0%) with an Aberdeen score <1 (*p* = 0.493).

## DISCUSSION

LGIB is a common clinical presentation associated with significant morbidity and mortality.^3^, ^18^ The present study shows the derivation, and internal and external validation of a prognostic scoring system to stratify risk in acute LGIB, showing for the first time that rebleeding and mortality have different risk factors. These scores use readily available clinical information and one blood test to calculate the risk of 30‐day rebleeding and mortality. Furthermore, low scores were associated with a very low risk of adverse outcomes and could support outpatient management. Perhaps the most important aspect is that this score can predict outcomes at presentation. Our score was superior to the Oakland score^11^ for predicting rebleeding but not mortality. The mortality score <1 was associated with a 0% 30‐day mortality in all cohorts and could be used in a similar way to the Blatchford score.^19^ The scoring system is easy to calculate and similar in format to other triaging systems.^6^, ^7^ The Oakland score, is not intuitive, has wide ranges for allocating scores allows risk stratification down to <5%, and requires a clinical decision meaning a patient can only be safely discharged if there is no other reason for admission. Therefore, even those scoring <8, may still require admission reflecting insufficient discrimination.

Previous studies show increasing age, hypotension, tachycardia, anemia, fresh blood on rectal examination, male sex, previous admission with LGIB, and active comorbidity are associated with increased risk of adverse outcomes.^8^, ^9^, ^10^, ^11^, ^20^, ^21^ Many are small (*n* < 500), used inappropriate modeling techniques, or used endoscopic databases for case identification rather than admission.^8^, ^10^ Some aimed to predict severe hemorrhage which is infrequent compared to rebleeding.^3^, ^11^, ^21^ One study investigated artificial neural networks to predict outcomes that performed well but were complex, costly, and difficult to interpret.^22^ The BLEED criteria appeared to have good predictive capacity but were not validated.^10^, ^20^ It might be expected that the underlying disease process would be associated with important outcomes in LGIB although this has not been shown in previous studies.^3^, ^11^ Interestingly, anticoagulants and/or antiplatelet agents were not incorporated into our scoring systems similar to the Oakland score.^11^ In the derivation cohort the main anticoagulant was warfarin as more recent agents such as rivaroxaban were not in clinical use. Both our cohort and the Oakland study did not examine the combination of anticoagulants and antiplatelet agents which might have a higher bleeding risk.

Recently, the ABC score has been developed to determine 30‐day mortality following either upper GIB or LGIB.^23^ This score only predicts mortality which occurred in 2.3% all of which were from the UK cohort.^3^ Comparison with this score was not possible as some variables were not collected in our cohorts.

Many of the variables in our score are in common with the Oakland score but there are differences in the variables’ strata. The Oakland score allocated risk scores even when in the normal range (e.g., systolic blood pressure 120–129 mmHg scores 3) whereas our score used recognized normal ranges. Also, the Oakland score recorded findings on rectal examination which the Bleeding Unit database did not record.

Our study could not characterize risk factors for transfusion and surgical intervention as these were not hard endpoints.

Our study has several strengths and limitations. The scores were derived from a large, prospectively collected database over 20 years and internally/externally validated using more contemporary years in two centers. Our score allows the risk of adverse events at 30 days to be reduced to <1% whereas the Oakland score used <5%.^11^

Neither our score nor the Oakland score has been applied prospectively to aid decision‐making or determine whether a specific intervention is needed, for example, blood transfusion.^11^ We only compared our score to the Oakland score as this appears superior to other scores.^11^ Our database was originally conceived many years ago, however, the management of LGIB in the UK has not changed and the validation cohorts were contemporary with similar adverse outcome rates supporting generalisability. It is possible that some patients were not captured in the database, but this number would be very low. All admissions with GI bleeding were directed to this unit with dedicated 24‐h access and staff who entered data. The inclusion of inpatients is potentially problematic as this is a heterogeneous group. Future exploration of such datasets should consider the exclusion of such patients. The population of Northeast Scotland is stable, and outcomes are tracked using electronic systems. Some patients had minor, self‐limiting bleeding not requiring admission representing very low risk. There was some missing data in the cohort with most variables having <5% missing but importantly, the outcome variables had <1% missing data. There were no patients in the highest strata of our scoring system, however, they would not be considered for discharge and management unaltered. Conversely, in the Oakland score derivation, no patients had some of the lowest scores suggesting this is difficult to obtain and the sensitivity of a low score may not be high.^11^, ^13^ One further limitation is separate scoring systems for the different adverse outcomes. However, we show that rebleeding and mortality have different risk factors in LGIB and this is reflected in the modelling. Given the most frequent adverse event is rebleeding, and the Aberdeen score is superior to the Oakland score for this, this may be the most practical use. Ideally, a unified scoring system that predicts these outcomes would be optimal.

The main issue in LGIB remains the lack of data showing intervention alters the risk of an adverse outcome. The UK national audit revealed a very low intervention rate and previous studies show no evidence that acute colonoscopy improves bleeding source identification, rebleeding, intervention, or surgery or affects mortality.^3^, ^14^, ^15^, ^16^ Irrespective of which score is used to triage patients, the choice of intervention is not clear. The most useful scoring system should identify patients at the lowest risk and support early discharge with outpatient management optimizing resource allocation. The ideal scoring system would determine which patients are most likely to require intervention. This is in stark comparison to upper gastrointestinal bleeding with well‐defined risk scoring systems and interventions shown to improve rebleeding and reduce the need for surgery, and death.^24^, ^25^

In summary, we have derived and validated a scoring system for patients with acute LGIB with good predictive value for adverse outcomes and is superior to the Oakland score for predicting rebleeding. A prospective head‐to‐head assessment of both scores is needed.

## CONFLICT OF INTEREST STATEMENT

None.

## Supporting information


**Document SA** Encoded values and descriptors for co‐morbidity gradations.

**Document SB** Factors associated with the need for blood transfusion and surgery within 30 days following admission with acute lower gastrointestinal bleeding.

**Document SC** Hosmer‐Lemershow test for final rebleeding multivariate model.

**Document SD** Hosmer‐Lemershow test for final mortality multivariate model.
