# Supplementary material for: Derivation, validation, and comparison of a new prognostic scoring system for acute lower gastrointestinal bleeding
Source: DEN Open. 2023 Dec 11;4(1):e323. doi: 10.1002/deo2.323 (PMC10713870; doi:10.1002/deo2.323)
Supplement: Supplementary file 1 — Document SA Encoded values and descriptors for co‐morbidity gradations. [file DEO2-4-e323-s002.docx]

# Supplementary document A

Encoded values and descriptors for co-morbidity gradations

| **Liver/GI** | **Score** |
| --- | --- |
| No disease | **0** |
| Inactive or mild liver disease/hepatitis, or controlled IBD | **1** |
| Cirrhosis/portal hypertension, moderate IBD, short bowel syndrome, chronic pancreatitis | **2** |
| Liver failure, encephalopathy, severe active IBD, acute pancreatitis | **3** |
| **Heart/Vascular** |  |
| No disease | **0** |
| Stable angina, old MI, hypertension, mild PVD | **1** |
| Bad angina, mild CCF, valvular heart disease or moderate PVD | **2** |
| Recent MI (last 4 weeks), severe heart failure, severe PVD | **3** |
| **Respiratory** |  |
| No disease | **0** |
| Mild asthma/COPD | **1** |
| Asthma/COPD chronic but no exacerbation, chest infection, PE (>2/12 ago) | **2** |
| Respiratory failure, PE (recent), pneumonia or bad exacerbation of asthma/COPD | **3** |
| **Arthritis** |  |
| No disease | **0** |
| Minor conditions, including mild Rh A, gout, problematic OA | **1** |
| Less severe Rh A etc., but requiring medication | **2** |
| Severe Rh A/connective tissue disease | **3** |
| **Stroke/Neurology** |  |
| No disease | **0** |
| Old stroke, Parkinsons on treatment or other chronic neurology | **1** |
| Stroke in last 6 months (with disability), recent TIA, severe dementia | **2** |
| Recent stroke (4wks) or severe neurological condition (severe Parkinsons) | **3** |
| **Renal** |  |
| No disease | **0** |
| Mild renal failure/impairment or renal disease | **1** |
| Chronic renal failure or renal disease | **2** |
| Acute renal failure, dialysis (haemo/peritoneal), transplant | **3** |
| **Malignancy** |  |
| No disease | **0** |
| Early/mild, chronic leukaemias and non-malignant cancers | **1** |
| Limited spread or treated cancer | **2** |
| Terminal cancer/widely disseminated cancer/acute leukaemias | **3** |
