# Supplementary material for: Derivation, validation, and comparison of a new prognostic scoring system for acute lower gastrointestinal bleeding
Source: DEN Open. 2023 Dec 11;4(1):e323. doi: 10.1002/deo2.323 (PMC10713870; doi:10.1002/deo2.323)
Supplement: Supplementary file 2 — Document SB Factors associated with the need for blood transfusion and surgery within 30 days following admission with acute lower gastrointestinal bleeding. [file DEO2-4-e323-s004.docx]

**Supplementary document B:** Factors associated with need for blood transfusion and surgery with 30 days following admission with acute lower gastrointestinal bleeding.

|  | **Blood transfusion** | **Surgical intervention** |
| --- | --- | --- |
| **Variable** | Odds ratio (95% confidence interval) | Odds ratio (95% confidence interval) |
| Age group  <60  60 - 79  ≥80 | 0.45 (0.34-0.59) p<0.001  Reference  1.74 (1.34-2.26) p<0.001 |  |
| Sex  Male  Female | Reference  1.88 (1.49-2.35) p<0.001 |  |
| Admission Source  Primary care  Secondary care |  | Reference  2.10 (1.44-3.07) p<0.001 |
| Melaena  No  Yes |  | Reference  1.69 (1.17-2.44) p=0.005 |
| Syncope  No  Yes |  | Reference  2.60 (1.36-4.96) p=0.004 |
| Other NSAID Use  No  Yes |  | Reference  1.62 (1.08-2.43) p=0.019 |
| Smoking  No  Yes |  | Reference  0.46 (0.28-0.77) p=0.003 |
| Arthritis grade  0  1  2  3 |  | Reference  0.45 (0.21-0.94) p=0.034  0.98 (0.30-3.23) p=0.979  1.22 (0.29-5.21) p=0.785 |
| Systolic BP  ≥100  <100 | Reference  4.52 (2.98-6.87) p<0.001 | Reference  2.37 (1.44-3.92) p=0.001 |
| Heart Rate  <100  ≥100 |  | Reference  1.51 (1.00-2.27) p=0.05 |
| Haemoglobin  ≥14  10-13.9  <10 | 0.13 (0.07-0.22) p<0.001  Reference  36.12 (24.84-52.52) p<0.001 | Reference  2.11 (1.28-3.45) p=0.003  3.02 (1.76-5.19) p<0.001 |
