# Supplementary figures and images for: Derivation, validation, and comparison of a new prognostic scoring system for acute lower gastrointestinal bleeding
Source: DEN Open. 2023 Dec 11;4(1):e323. doi: 10.1002/deo2.323 (PMC10713870; doi:10.1002/deo2.323)

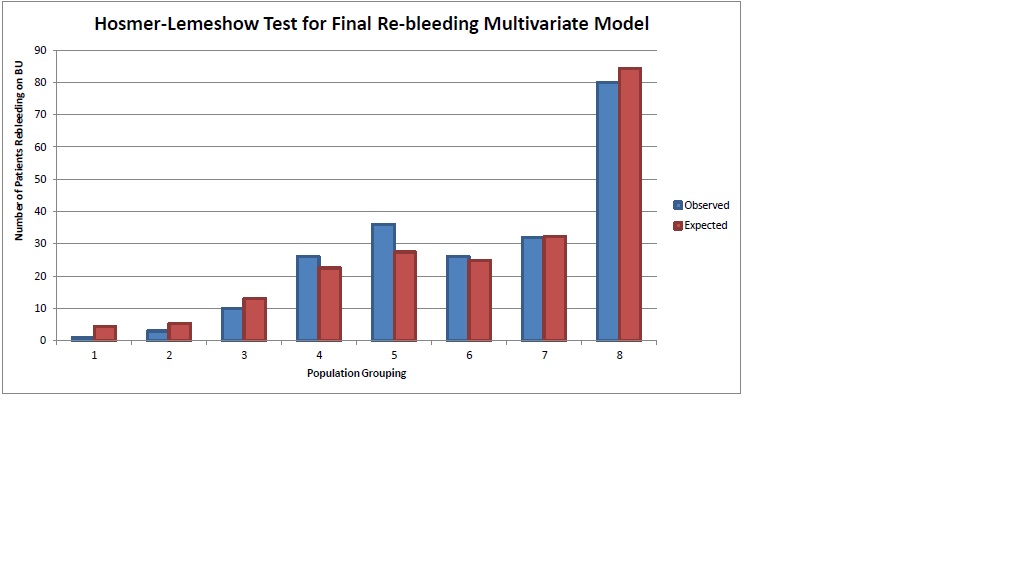

Supplement: Supplementary file 3 — Document SC Hosmer‐Lemershow test for final rebleeding multivariate model. [file DEO2-4-e323-s003.jpg]

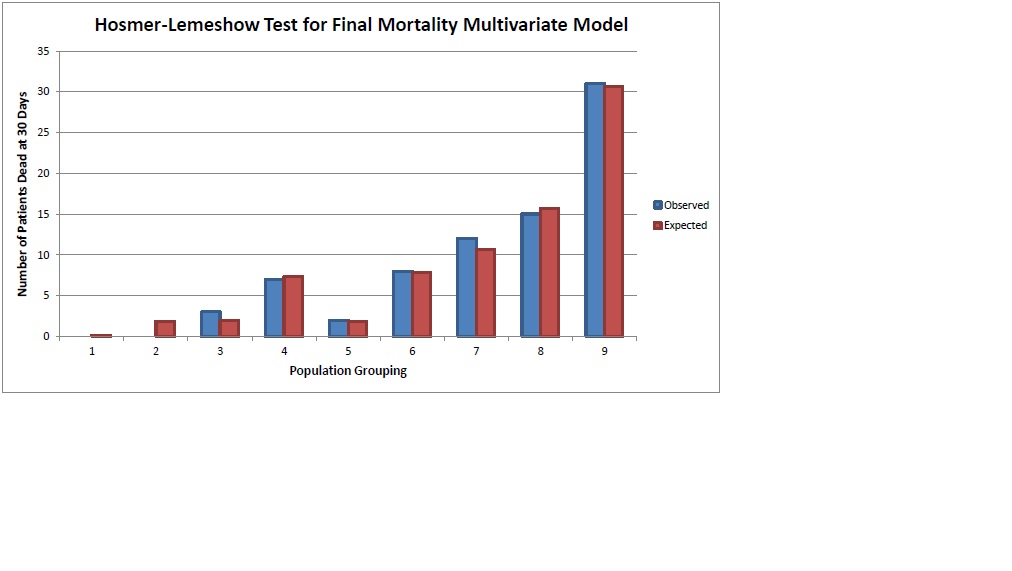

Supplement: Supplementary file 4 — Document SD Hosmer‐Lemershow test for final mortality multivariate model. [file DEO2-4-e323-s001.jpg]
